# Supplementary material for: Self-Restoring Capacitive Pressure Sensor Based on Three-Dimensional Porous Structure and Shape Memory Polymer
Source: Polymers (Basel). 2021 Mar 8;13(5):824. doi: 10.3390/polym13050824 (PMC7962847; doi:10.3390/polym13050824)
Supplement: Supplementary file 1 [file polymers-13-00824-s001.pdf]

Supporting Information

Self-Restoring Three-dimensional Porous Structure Capacitive Pressure Sensor Using Shape Memory Polymer

Byung-geon Park[1, 2, ‡], Young Jung[1,2, ‡], Jong Soo Ko[2], Jinhyoung Park[3]* and Hanchul Cho[1,*]

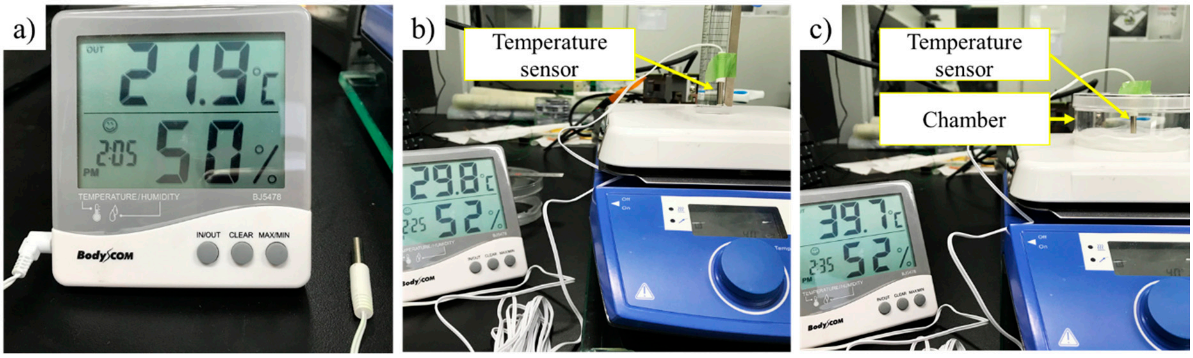

Figure S1. Specially designed chamber to maintain 40 °C. (**a**) Room temperature. (**b**) Sensor without the chamber at approximately 30 °C when the hotplate is set at 40 °C. The temperature sensor is fixed at the same height as the chamber by using a ruler. (**c**) In the chamber, the temperature is approximately 40 °C when the hotplate is set at 40 °C.

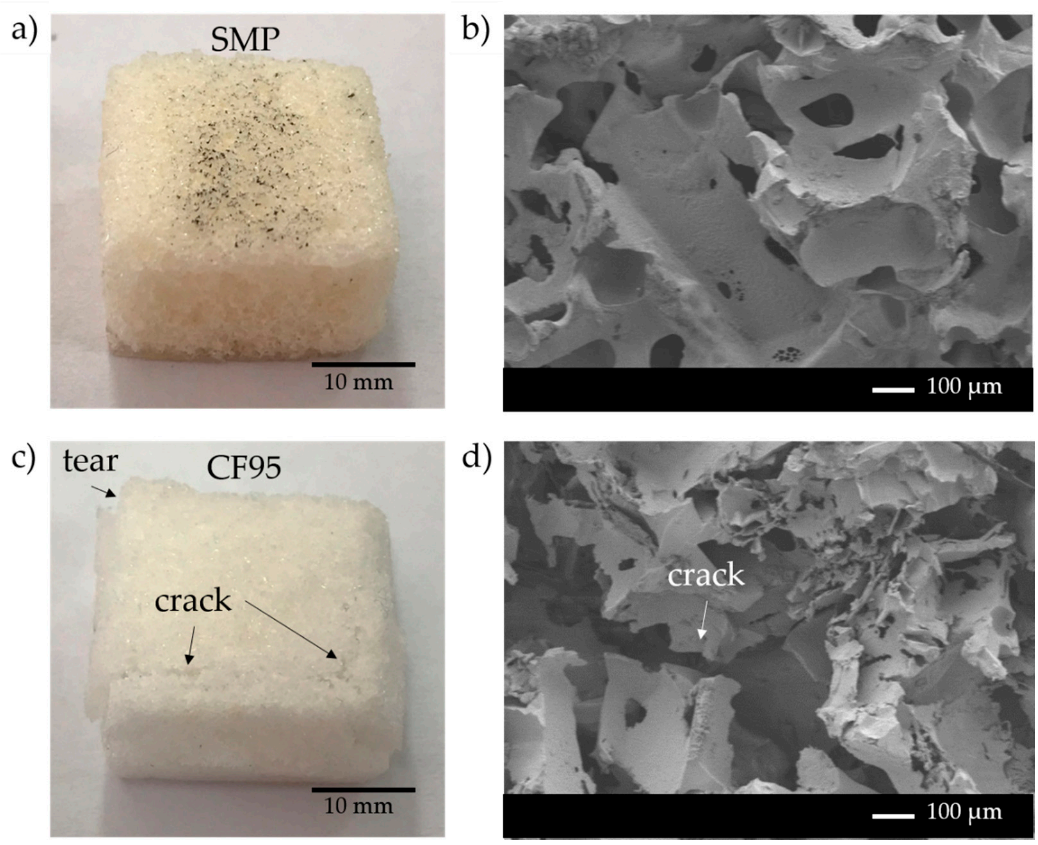

Figure S2. Structural images of the SMP and CF95 porous structures (all images are after the 100-cycle loading test). (**a**) Photograph of the SMP porous structure: no significant change to the structure is observed. (**b**) SEM image of the SMP porous structure. (**c**) Photograph of the CF95 porous structure: some cracks and tears are evident on the structure. (**d**) SEM image of the CF95 porous structure: microcracks are evident in the structure.

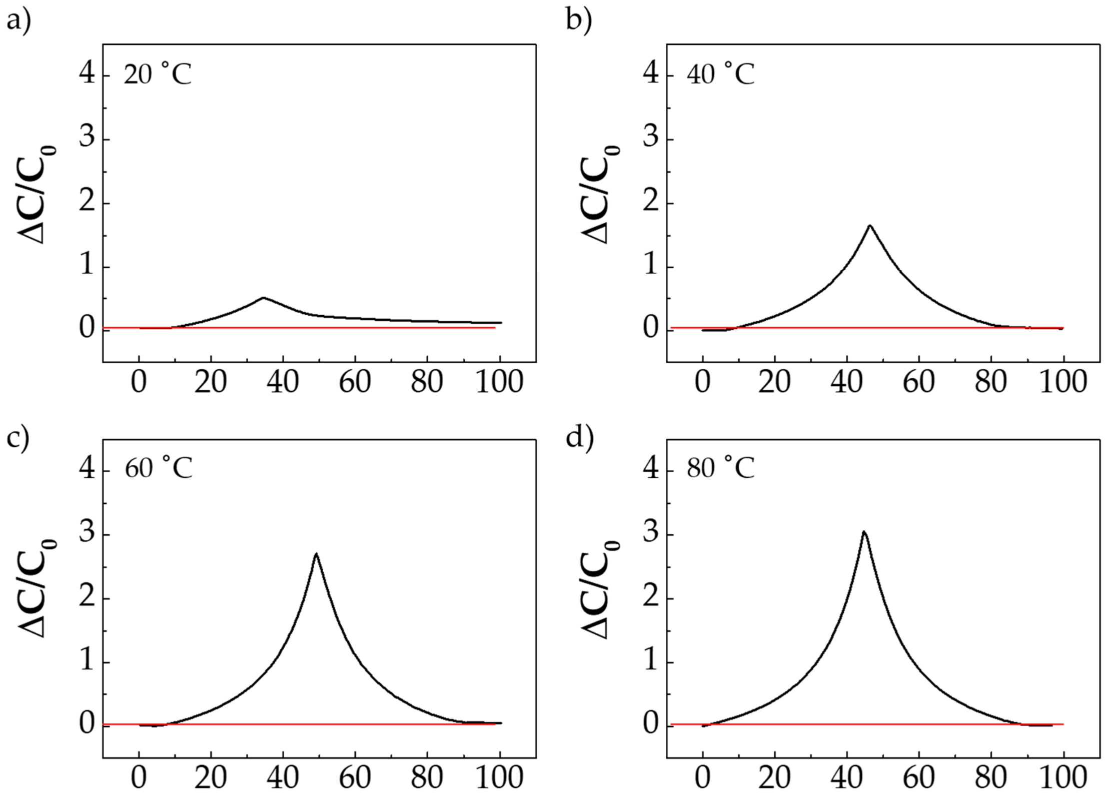

Figure S3. SMP porous structure sensor performances at 500 kPa according to temperature. (a) Sensor performance at 20 °C: without recovery ability. It cannot recover to initial state. (b) Sensor performance at 40 °C: the response of sensor is perfectly symmetric, and the sensitivity at the same compressive load is more than 3 times higher than it at 20 °C. (c) Sensor performance at 60 °C: the response of sensor is similar with the result at 40 °C. (d) Sensor performance at 80 °C.
